# Comparison of clinical efficacy of different internal fixation methods in older adult patients with osteoporotic fractures of proximal humerus

**DOI:** 10.3389/fsurg.2024.1472054

**Published:** 2025-01-15

**Authors:** Zongpu Wang, Tienan Wang, Song Qin, Jianchuan Wang

**Affiliations:** ^1^ Department of Orthopedics, Affiliated Zhongshan Hospital of Dalian University, Dalian, China; ^2^ School of Mechanical Engineering, Dalian Jiaotong University, Dalian, China

**Keywords:** fracture of proximal humerus in older adult, osteoporosis, internal fixation with plates, half-shoulder joint replacement, calcium sulfate

## Abstract

**Objective:**

To compare the efficacy of three treatment methods for older adult patients with osteoporotic proximal humerus fractures: proximal humerus locking plate (PHILOS) combined with calcium sulfate injection, PHILOS plate alone, and artificial hemi-shoulder joint replacement.

**Methods:**

The clinical data of 48 older adult patients with osteoporotic proximal humerus fractures admitted to the Shoulder and Elbow Surgery Department of Zhongshan Hospital Affiliated with Dalian University from February 2018–August 2021 were retrospectively analyzed. The patients comprised 18 males and 30 females, with a mean age of (68.6 ± 5.8) years. The 48 patients were divided into three groups based on their treatment methods: Group A: 16 patients treated with PHILOS plate combined with calcium sulfate injection. Group B: 16 patients treated with the PHILOS plate alone. Group C: 16 patients treated with artificial hemi-shoulder joint replacement. Key parameters such as operation time, blood loss, incision length, operation cost, and shoulder joint motion at the final follow-up were recorded and compared. Shoulder joint function was evaluated using the American Shoulder and Elbow Surgeons (ASES) score, Visual Analogue Scale (VAS) for pain, University of California Shoulder Joint Score (UCLA), and Brief Shoulder Function Test (SST).

**Results:**

There were no statistically significant differences in the preoperative general data between the three groups (*P* > 0.05), indicating comparability. There was no significant difference in operation time, blood loss, and incision length among Groups A, B, and C (*P* > 0.05). However, Group C had significantly higher operation costs compared to the other two groups (*P* < 0.05). At the final follow-up, there were no significant differences in shoulder flexion and rotation among the three groups (*P* > 0.05). However, a statistically significant difference in abduction was observed between Group A and Group C (*P* < 0.05). No significant differences were found in ASES, VAS, UCLA, and SST scores among the three groups at the last follow-up (*P* > 0.05).Complications occurred in 1 patient (Group A), 3 patients (Group B), and 4 patients (Group C), showing a statistically significant difference among the groups (*P* < 0.05).

**Conclusion:**

All three surgical methods are effective in treating older adult osteoporotic proximal humerus fractures, as they significantly alleviate pain and restore joint function. However, the use of a PHILOS plate combined with calcium sulfate injection (Group A) is particularly effective, demonstrating reliable clinical efficacy with fewer complications.

## 1 Introduction

Osteoporosis is a systemic metabolic bone disease characterized by pain, bone deformation, and susceptibility to fractures. Osteoporotic patients are often afflicted with comorbid conditions, such as cardiovascular diseases, diabetes, and neurodegenerative disorders. Over the past two decades, the global incidence of osteoporosis has reached approximately 18.3%. Proximal humerus fractures are the second most common upper-limb fractures after distal radius fractures, accounting for 10% of all fractures in individuals over 65 years of age and 17.5% of osteoporotic fractures in postmenopausal women over 50 years old (1). With an aging global population, the incidence of proximal humerus fractures is increasing and is projected to triple by 2030 (2).The severity of displacement and the likelihood of complications increase with age, particularly in large-displacement three- or four-part fractures. Such fractures result in elevated medical costs, functional impairment, and reduced quality of life. Conservative treatment may suffice for fractures with minimal displacement; however, for complex fractures, conservative management can lead to malunion, nonunion, stiffness, and persistent pain (3, 4).Current treatments for osteoporosis include calcium, vitamin D, bisphosphonates (e.g., alendronate and risedronate), and alternative therapies such as traditional Chinese medicine and massage. Surgical techniques, including percutaneous fixation, intramedullary nailing, and joint replacement, have been developed to address complex fractures (5). Open reduction and internal fixation using locked plates has gained widespread acceptance, yielding favorable clinical outcomes. However, complications such as screw loosening, humeral head necrosis, and fixation failure remain prevalent, particularly in older adult patients with poor bone quality, with complication rates as high as 34% (6, 7).To address these challenges, recent research has explored methods to enhance screw-bone interface strength, such as intramedullary fibular bracing and the use of bone cement (8, 9). This study retrospectively analyzed the clinical data of older adult patients treated with PHILOS plates combined with calcium sulfate, PHILOS plates alone, and hemi-shoulder joint replacement. The aim was to compare the efficacy and complication rates among these three treatment modalities.

## 2 Materials and methods

### 2.1 Inclusion and exclusion criteria

Inclusion criteria: (i) According to the Neer classification, the proximal humerus fractures were either three-part or four-part fractures. (ii) Patients with osteoporotic fractures of the proximal humerus treated in our hospital from February 2018–August 2021. (iii) Unilateral fresh limb fracture, with no disease of the shoulder joint before injury. (iv) The patient and his or her family agree on the treatment plan. (v) Complete follow-up of clinical data.

Exclusion criteria: (i) Pathological fracture. (ii) Open fracture. (iii) Multiple fractures of the same limb, with obvious dysfunction of the shoulder joint before injury. (iv) Patients and their families refuse surgery. (v) Incomplete follow-up of clinical data.

### 2.2 General information

A total of 48 patients meeting the inclusion criteria were analyzed (18 males, 30 females), with a mean age of (68.6 ± 5.8) years (range: 60–81 years). The injuries were primarily caused by falls, impacts, and motor vehicle accidents. x-ray, CT, and MRI examinations were performed preoperatively. All procedures were completed within 7 days post-injury by the same surgical team. The patients were divided into three groups: Group A: PHILOS plate combined with calcium sulfate injection (16 cases: 10 three-part, 6 four-part fractures). Group B: PHILOS plate alone (16 cases: 9 three-part, 7 four-part fractures). Group C: Hemi-shoulder joint replacement (16 cases: 7 three-part, 9 four-part fractures).

### 2.3 Surgical techniques

All eligible patients underwent general anesthesia, were placed in the supine position with the shoulder on the affected side elevated. Routine disinfection was performed in group A and group B, and iodine tincture disinfection was done in group C and for all surgical participants. Sterile sheets and films were applied in the surgical area.

**Figure 1:**
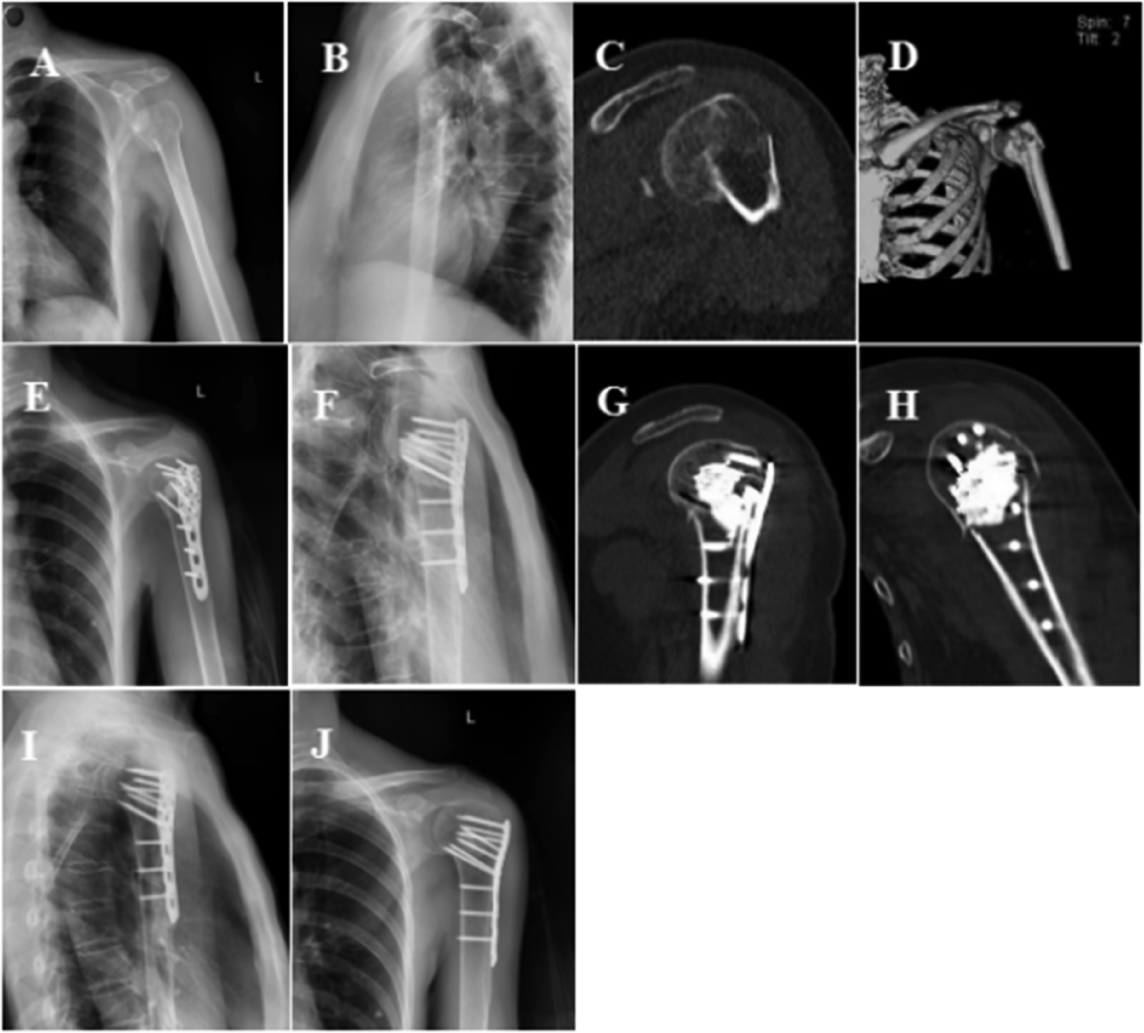
Follow-up imaging of comminuted fracture of proximal left humerus. **(A,B)** Preoperative X-rays showed fracture displacement, humeral head inversion, and displacement of large and small tubercles. **(C,D)** Preoperative CT scan of the left shoulder showed fracture inversion, separation and displacement of large and small nodules. **(E,F)** Postoperative x-rays showed that the fracture was well reduced and the humerus head was adequately filled with calcium sulfate injection. **(G,H)** Postoperative shoulder joint CT showed that injection of calcium sulfate filled the humerus skull defect adequately. **(I,J)** At the last follow-up, the fracture healed and calcium sulfate was completely degraded and absorbed.

Group A: The surgical approach was the deltoid and pectoralis major approach. Blunt separation was used to expose the cephalic vein, with emphasis on protection to avoid damage to the axillary nerve and the long head tendon of the biceps. The insertion of the deltoid muscle was partially severed, and the broken end of the fracture was fully exposed. The fracture was reduced and temporarily fixed with the cross of a Kirschner's needle. The locking plate was placed behind the intertubercular sulcus and 0.5 cm below the apex of the greater tubercle. A lag screw was temporarily fixed in a non-locking hole. C-arm X-ray was used to determine the appropriate height and position of the steel plate. Then, the locking screw and the humerus talus screw of sufficient length were screwed in respectively. The middle nail hole of the locking plate was chosen, and the prepared calcium sulfate was evenly injected into the humerus head and the defect of the humerus talus using a calcium sulfate injector. At the same time, the injection was pumped back into the injector until it could not be injected. Finally, the position of the steel plate, the length of the screw, and the filling of calcium sulfate were re-examined under the C-arm. The rotator cuff was repaired by suture. The rotator cuff was rinsed repeatedly, and the passive shoulder joint had good motion. A drainage tube was placed under the skin, and the incision was closed layer by layer (Figure 1).

Group B: The deltoid-pectoralis major space approach was used to incise the skin and bluntly separate it to the deep. The head vein and the long head tendon of the biceps were protected, and the deltoid-muscle insertion was bluntly peeled to fully expose the humerus head and fracture block. The humerus head and fracture block were fully exposed, and the humerus head and fracture block were reduced under direct vision, and a Kirschner's needle was used for temporary fixation. The locking plate was placed in the anterolateral internodal groove at 0.5 cm. The position of the plate was determined by C-arm fluoroscopy. Locking screws of appropriate length were screwed in turn. The fractures were re-reduced by fluoroscopy, and the steel plate height was appropriate. The rotator cuff and soft tissue were sutured, hemostasis was fully achieved, drainage tubes were thoroughly rinsed, and the incision was sutured.

Group C: The deltoid-pectoralis space approach was taken to protect the head vein, axillary nerve, and rotator cuff tissue, fully expose the proximal end of the humerus, mark the humerus osteotomy plane and perform osteotomy, expand the medullary canal, irrigate, reduce the prosthesis test model, locate the X-ray prosthesis test model, determine the prosthesis height and retrograde angle of the humerus head, remove the test model, fill the medullary cavity with bone cement, and select the appropriate prosthesis for installation. After the bone cement hardened and the shoulder joint was reduced, the large and small nodules were reconstructed, the rotator cuff was repaired, full hemostasis was achieved, and the incision was closed.

A 68- year-old female patient suffered a comminuted fracture of the proximal left humerus (Neer type IV) caused by a fall. (A, B) Preoperative anterolateral X-rays of the left shoulder showed a proximal humerus fracture, varus involvement of the fracture in the medial humerus talus, displacement of large and small tubercle fractures, and an increased retrograde humerus head; (C, D) Left-shoulder CT and 3D reconstruction clearly showed fracture inversion, involvement of the humerus talus, prominence, separation, and displacement of the large and small tubercles; (E, F) Post-operative X-ray results showed anatomic reduction of the fractures, correction of the varus, support of the humerus talus screw for varus, and the injectable calcium sulfate fully filled the humerus head and the defect of the humerus talus; (G, H) Sagittal and coronal CT images of the proximal humerus of the left shoulder after surgery showed sufficient calcium sulfate implantation in the humerus head and the bone defect within the humerus talus, and the screws were evenly surrounded by calcium sulfate. (I, J) Three months after surgery, the left shoulder was re-examined in the anterolateral position, showing fracture healing, complete degradation of calcium sulfate, and fusion of normal bone tissue.

### 2.4 Postoperative management and evaluation of curative effect

After the operation, antibiotics were routinely administered for 3 days to prevent infection. In group A and group B, the shoulder and elbow straps were suspended and fixed. Passive pendulum—type functional exercises were performed 2 weeks after the operation to avoid shoulder joint adhesion. Passive abduction, forward flexion, and backward extension activities were started 3 weeks after the operation, and full shoulder joint activities were gradually carried out 4–6 weeks after the operation. In group C, an abduction brace was fixed. To avoid the rotator cuff pulling the greater tubercle and resulting in greater tubercle displacement, passive functional exercises were performed 2 weeks after the surgery, and shoulder joint functional exercises were gradually started 3 weeks after the surgery. Anterolateral X-rays of the shoulder joint were periodically reviewed at 2, 4, 8, 12, 6, and 12 weeks after the surgery to evaluate fracture healing and internal fixation.

The operative time, blood loss, incision length, and surgical cost of the three groups were compared, and the shoulder joint motion at the last follow-up was recorded. The shoulder joint function was evaluated using the American Shoulder and Elbow Surgical Score (ASES), the visual analogue pain score (VAS), the University of California Shoulder Joint Score (UCLA), and the Brief Shoulder Joint Function Test score (SST).

### 2.5 Statistical analysis

Statistical analysis was performed using IBM SPSS 26.0 software. The Shapiro-Wilk test was applied to assess the normality of the data distribution. Measurement data conforming to normality and homogeneity of variance were expressed as mean ± standard deviation (*x* ± s). The chi-square (*χ*²) test was used to compare categorical data. A *P*-value of <0.05 was considered statistically significant.

## 3 Results

here were no statistically significant differences in gender, age, injured limb, time to surgery, or Neer classification among Groups A, B, and C (*P* > 0.05), confirming comparability (Table 1). All patients underwent surgery successfully, with primary healing of surgical incisions and no incidences of infection. The mean follow-up duration was (16.5 ± 2.6) months.

**Table 1:** Basic data of three groups of patients (*n* = 16).

Group	Sex (case)		Age	Sidelong (case)		Waiting time	Neer type	
	Male/female		(year, $\bar{x}\pm s$)	Left right		(*d*, $\bar{x}\pm s$)	III/IV	
A group	7	9	69.2 ± 10.6	6	10	4.2 ± 1.5	10	6
B group	6	10	68.6 ± 12.3	11	5	4.0 ± 1.8	9	7
Cgroup	8	8	70.7 ± 9.8	9	7	4.1 ± 2.0	7	9
*t* value	2.323		0.537	1.023		0.054	3.453	
*P* value	0.25		0.432	0.391		0.840	0.079	

Patients in group A, group B and group C had no statistical significance in terms of operation time, blood loss and incision length (*P* > 0.05), but the operation cost in group C was higher than that in the other two groups, and the pairings were statistically significant (*P* < 0.05) (Table 2).

**Table 2:** Comparison of operation time, blood loss, incision length and operation cost among three groups (*x* ± s, *n* = 16).

Group	Operation time	Blood loss volume	Incision length	Operation cost
	(min)	(ml)	(cm)	(yuan)
A group	98.00 ± 10.23	210.81 ± 10.36	10.8 ± 1.2	27,791 ± 2,300
B group	95.12 ± 9.86	204.26 ± 15.61	10.5 ± 2.0	23,012 ± 3,900^a^
C group	90.23 ± 12.65	205.10 ± 12.42	11.3 ± 1.8	42,031 ± 4,100^a^^,^^b^
*t* value	15.331	0.965	2.524	7.879
*P* value	0.534	0.645	0.251	<0.001

Note: Group A was treated with proximal humerus PHILOS plate combined with calcium sulfate injection. Group B was treated with proximal humerus PHILOS plate alone. Group C was treated with artificial half-shoulder joint replacement. The comparison between group *a* and group A was <0.05, and the comparison between group *b* and group B was <0.05.

^a,b^Represent comparison with group A and group B.

There was no significant difference in anterior flexion and external rotation of shoulder between groups A, B and C at the last follow-up (*P* > 0.05). Outreaching activity: The difference between group A and group C was statistically significant (*P* < 0.05) (Table 3).

**Table 3:** Comparison of shoulder joint flexion, external rotation and abduction motion between the three groups to the last follow-up (*x* ± s, *n* = 16).

Group	Last follow-up	Last follow-up	Last follow-up
	Shoulder joint forward bend (°)	External rotation of shoulder joint (°)	Shoulder joint abduction (°)
A group	135.5 ± 6.2	40.1 ± 4.9	138.1 ± 12.1
B group	132.2 ± 5.3	38.8 ± 5.6	130.6 ± 11.7
C group	130.1 ± 2.8	37.3 ± 3.4	120.2 ± 10.2^a^
*t* value	0.607	2.112	3.423
*P* value	0.235	0.183	0.064

Note: Group A was treated with proximal humerus PHILOS plate combined with calcium sulfate injection. Group B was treated with proximal humerus PHILOS plate alone. Group C was treated with artificial half-shoulder joint replacement. The comparison between group C and group A was <0.05, and the comparison between group C and group B was <0.05.

^a^Represents the comparison between group C and group A

At the last follow-up, there were no significant differences in ASES score, VAS pain score, UCLA score and SST score among the three groups (*P* > 0.05). (Table 4) Complications of A, B, and C: 1 case in group A showed extravasation of injected calcium sulfate, which was absorbed by itself during follow-up. In group B, 1 screw was cut out, 1 screw was inverted, and 1 screw was loosened. In group C, there were 2 cases of macrotuberous absorption and 2 cases of joint stiffness. The difference among the three groups was statistically significant (*P* < 0.05).

**Table 4:** Comparison of ASES scores, VAS pain scores, UCLA scores and SST scores in the three groups from the last follow-up (*x* ± s, *n* = 16).

Group	ASES score	VAS score	UCLA score	SST score
A group	85.2 ± 4.3	1.1 ± 0.5	31.0 ± 1.2	8.6 ± 0.8
B group	84.5 ± 3.2	1.0 ± 0.4	30.2 ± 1.1	7.9 ± 0.9
C group	83.3 ± 3.5	1.0 ± 0.7	29.9 ± 0.9	7.8 ± 1.0
*t* value	0.426	0.189	1.021	0.653
*P* value	0.501	0.842	0.963	0.231

## 4 Discussion

Treatment options for proximal humerus fractures in older adults, especially osteoporotic fractures, are challenging, as these fractures account for 6% of adult fractures and their incidence continues to increase with age, as the majority of the population occurs over the age of 65 (10). At present, osteoporotic fractures of the proximal humerus in the older adult have always been a difficult problem for orthopedic surgeons in clinical practice, because fractures of the proximal humerus can lead to severe pain and loss of function of the shoulder joint, which seriously affects the quality of life. The treatment options include conservative treatment, open reduction and internal fixation, joint replacement, etc (11). However, there is no consensus on the treatment of 3 or 4 partial fractures of the proximal humerus, and each option has its own advantages and disadvantages, so there is a lack of management consistency in the selection of treatment options. Regardless of the treatment, the primary goal for this fracture is to promote uncomplicated bone healing to re-establish a painless mobility, stability and function of the shoulder joint.

Proximal humerus locking plate (PHILOS) is an anatomical locking plate designed by AO/OTA to improve functional outcomes, especially in patients with osteoporosis. The locking capability of the screw provides better locking of osteoporotic bone to the plate, gives the structure angular stability, and maintains postoperative reduction during early functional rehabilitation, avoiding joint stiffness. It has become the implant of choice for internal fixation of proximal humerus fractures (12). Despite this, the incidence of complications in open reduction and internal fixation is still high, Thanasas et al. (13). Results showed that lock-plate internal fixation was associated with fracture displacement (12.2%), screw removal (11.6%), fracture nonunion (13%), and avascular necrosis (7.9%). Therefore, people gradually realized the importance of these complications, and treatment strategies began to focus on the internal structure of the humerus head, such as bone cement reinforcement and bone graft support to avoid reducing the incidence of complications (14, 15). Schultz et al. (16) applied PHILOS plate combined with fibula support to treat proximal humeral fracture patients with medial talar column fracture, and this method showed that it could reduce internal fixation complications and obtain good clinical results. However, for patients with varus angulation, two - and three-part proximal humeral fractures, there was no statistical significance in the clinical effect of the addition of a fibula brace vs. the absence of a fibula brace, and for older adult patients with osteoporotic fractures of the proximal humerus, the use of an autofibula brace, if used, was not statistically significant. Older adult patients have poor bone conditions and increased surgical trauma, so the clinical promotion of this treatment plan has certain limitations (17). Researchers began to fill the bone defect by implanting filler into the humerus head to increase the screw grip and accelerate bone healing (18). The commonly used filler bone cement has a good biological type advantage. VanVeelen et al. (19) used hollow bone cement to strengthen screw fixation in osteoporotic fractures of the proximal humerus, and the results showed that it could reduce the complications of internal fixation failure such as screw loosening and removal. However, the temperature of PMMA during the polymerization stage can be as high as 100°C, which may lead to bone and cartilage necrosis (20). In view of the above situation, more solutions should be sought. It has been reported that shoulder joint replacement for osteoporotic fractures of the proximal humerus can achieve good clinical efficacy and effectively relieve the pain symptoms of patients, but the prognosis is also different. If the greater tubercle can achieve anatomic reduction and healing, good function can be achieved. The incidence of non-union of the greater tubercle is higher in older adult patients, which directly affects shoulder joint function and correspondingly reduces shoulder joint score (21, 22).

In the face of internal bone defects in the humerus head, resulting in decreased screw control, autologous cancellous bone transplantation is the gold standard, but there are limitations such as donor site morbidity and insufficient filling. Currently, there are many substitute bone transplantation materials, each with its own shortcomings, limiting clinical application. Calcium sulfate is an early bone graft material with good self-consolidation, shape ability, and degradation absorption rate close to the new bone formation rate. During absorption, a large number of bone trabeculae and bone marrow are formed and connected to each other to form a structure similar to normal bone tissue. The remaining calcium sulfate continues to play the role of bone conduction scaffold, and the residual or exudated calcium sulfate in the joint space is absorbed within 15 days. It has advantages over other bone graft materials (23, 24). Based on the above advantages of calcium sulfate, our team used PHILOS combined with injectable calcium sulfate to treat older adult patients with osteoporotic fractures of the proximal humerus. Calcium sulfate was injected into the bone defect of the humerus head to increase the bone mass in the head, improve the screw holding ability, and finally calcium sulfate degradation fused with normal bone tissue. Satisfactory clinical efficacy was achieved through clinical follow-up. It provides a new treatment method for senile proximal humerus osteoporotic fracture.

## 5 Conclusions

In summary, for the treatment of older adult proximal humerus osteoporotic fractures, the proximal humerus locking plate (PHILOS) combined with calcium sulfate injection, the simple proximal humerus locking plate, and artificial half-shoulder replacement can effectively relieve pain and restore joint function. However, the proximal humerus locking plate (PHILOS) combined with calcium sulfate injection has fewer complications, costs less than artificial half-shoulder replacement, and has better abductor motion than joint replacement. Moreover, its clinical efficacy is reliable, making it one of the effective options for older adult proximal humerus osteoporotic fractures. Nevertheless, in clinical practice, the choice of surgery requires clinicians to make a comprehensive consideration based on the fracture type, surgical indications, and the individual patient.

## 6 Limitations

There are some limitations in this study: (i) The number of studies and patients is small, so more sample size needs to be included; (ii) All the included samples were admitted to our hospital and were single-center studies, so the results may be biased and need to be verified by large-sample multi-center studies; (iii) The follow-up time is short, and long-term follow-up is needed to observe the clinical effect; (iv) This study is a retrospective case-control study, and there may be some selective bias in the selection of cases, which needs to be verified by a large sample randomized controlled study.

## Data Availability

The datasets presented in this study can be found in online repositories. The names of the repository/repositories and accession number(s) can be found in the article/Supplementary Material.
